# Supplementary material for: Polyketide synthase gene clusters in the ericoid mycorrhizal fungus Oidiodendron maius: genome-wide prediction, expression profiling, and functional characterization of knockout mutants
Source: Front Plant Sci. 2026 Sep 16;17:1924430. doi: 10.3389/fpls.2026.1924430 (PMC13623903; doi:10.3389/fpls.2026.1924430)
Supplement: Supplementary file 1 [file SupplementaryFile1.zip › Supplementary Materials/Supplementary Folder 1.zip!/Oidma1_AssemblyScaffolds_Repeatmasked/knownclusterblast/cluster50/jgi.p_Oidma1_172955_mibig_hits.html]

| MIBiG Protein | Description | MIBiG Cluster | MiBiG Product | % ID | % Coverage | BLAST Score | E-value |
| --- | --- | --- | --- | --- | --- | --- | --- |
| AMY15055.1 | MFS\_transporter | BGC0001339 | Squalestatin\_S1\_biosynthetic\_gene\_cluster | 34 | 96.7741935484 | 306 | 5.3e-83 |
| BAE56594.1 |  | BGC0001123 | Aspirochlorine\_biosynthetic\_gene\_cluster | 32 | 96.0573476703 | 241 | 2.1e-63 |
| ACS68556.1 | major\_facilitator\_superfamily\_protein | BGC0001026 | NG-391\_biosynthetic\_gene\_cluster | 34 | 78.1362007168 | 236 | 8.8e-62 |
| AAB36607.1 | toxin\_pump | BGC0000370\_c2 | HC-toxin\_biosynthetic\_gene\_cluster | 25 | 91.5770609319 | 149 | 1.1e-35 |
| AFU65888.1 | DacR2 | BGC0000216 | Dactylocycline\_biosynthetic\_gene\_cluster | 26 | 94.623655914 | 142 | 1.3e-33 |
| AAS90069.1 | AflT | BGC0000010 | Aflatoxin\_biosynthetic\_gene\_cluster | 25 | 90.3225806452 | 130 | 6.8e-30 |
| BAE71313.1 | putative\_ABC\_transporter | BGC0000004 | Aflatoxin\_biosynthetic\_gene\_cluster | 25 | 79.0322580645 | 127 | 5.8e-29 |
| AAS90046.1 | AflT | BGC0000009 | Aflatoxin\_biosynthetic\_gene\_cluster | 26 | 90.3225806452 | 125 | 2.2e-28 |
| AAS89998.1 | AflT | BGC0000007 | Aflatoxin\_biosynthetic\_gene\_cluster | 25 | 89.9641577061 | 124 | 2.9e-28 |
| AAS90021.1 | AflT | BGC0000008 | Aflatoxin\_biosynthetic\_gene\_cluster | 25 | 90.1433691756 | 123 | 8.3e-28 |
| AAS90092.1 | AflT | BGC0000006 | Aflatoxin\_biosynthetic\_gene\_cluster | 25 | 89.9641577061 | 123 | 1.1e-27 |
| ACD39765.1 | major\_facilitator\_superfamily\_transporter | BGC0000077 | Hypothemycin\_biosynthetic\_gene\_cluster | 26 | 89.247311828 | 117 | 4.6e-26 |
| ACD39756.1 | major\_facilitator\_superfamily\_transporter | BGC0000076 | Hypothemycin\_biosynthetic\_gene\_cluster | 26 | 89.247311828 | 117 | 4.6e-26 |
| AGC24258.1 | PrlD | BGC0001038 | Pyralomicin\_biosynthetic\_gene\_cluster | 24 | 74.1935483871 | 96 | 1.1e-19 |
| EYT83437.1 | multidrug\_MFS\_transporter | BGC0001213 | Nataxazole\_biosynthetic\_gene\_cluster | 25 | 75.0896057348 | 92 | 1.2e-18 |
| ACN69979.1 | efflux\_transporter | BGC0000079 | Indanomycin\_biosynthetic\_gene\_cluster | 26 | 79.3906810036 | 91 | 3.5e-18 |
| CAQ64681.1 | putative\_resistance\_protein,\_transporter | BGC0000087 | Lasalocid\_biosynthetic\_gene\_cluster | 24 | 86.917562724 | 88 | 3e-17 |
| BAG85020.1 | putative\_resistance\_protein | BGC0000086 | Lasalocid\_biosynthetic\_gene\_cluster | 24 | 86.917562724 | 88 | 3e-17 |
| ctg1\_orf0002 |  | BGC0000688 | Copalyl\_diphosphate\_biosynthetic\_gene\_cluster | 26 | 65.4121863799 | 87 | 6.6e-17 |
| CAH60162.2 | putative\_transmembrane\_efflux\_protein | BGC0000700 | Istamycin\_biosynthetic\_gene\_cluster | 25 | 76.523297491 | 86 | 8.7e-17 |
| ABL09967.1 | transmembrane\_efflux\_protein | BGC0000197 | Aranciamycin\_biosynthetic\_gene\_cluster | 25 | 75.0896057348 | 86 | 1.1e-16 |
